# Introductory Address Delivered at the Commencement of the Session of 1862–3 of Rush Medical College

**Published:** 1862-11

**Authors:** J. Adams Allen

**Affiliations:** Professor of Principles and Practice of Medicine, and Clinical Medicine


CHICAGO
MEDICAL JOURNAL.

VOL. VI.] NOVEMBER, 1862. [No. 11.

### ORIGINAL COMMUNICATIONS.

### INTRODUCTORY ADDRESS

DELIVERED AT THE COMMENCEMENT OF THE SESSION OF 1862-3 OF RUSH MEDICAL COLLEGE,

By J. ADAMS ALLEN, M. D., LL. D.,

*Professor of Principles and Practice of Medicine, and Clinical Medicine.*

Off the coast of "the fast anchored isle" rises from the sea a range of ragged rocks which the tide at its ebb and flow alternately covers and discloses. When the storm rules the ocean, the spectacle presented is of most fearful grandeur. The waves break upon the reef with a power which appals the stoutest-hearted mariner.

It happens that this spot is precisely in the track of England's greatest maritime traffic. For ages it had been the terror of all those who go down to the sea in ships. Wo to those whom the darkness or the storm, or a trifling error in nautical calculations, brought into its neighborhood. Despairing signals hung out from tall masts availed nothing. Melancholy minute guns brought no answer save their own hoarse reverberations. The skeletons of massive men-of-war and peaceful merchantmen, alike lined the distant shore with their ribs of English oak.

Upon the uttermost verge of this dangerous shoal, national enterprise, by and by, planted a light-house. A little while it stood, but the great storms of autumn came and swept it away. It was replaced again, and again, and again, until the hearts of the majority failed them, and the attempt was desisted from. But there came a man who said the project might yet succeed—that all that was needed for success was to avoid the errors of the past, and adapt the means to ends — to meet power by power, and, where the contest was unequal, to meet it by thoughtful invention such as clear human heads had abundance of. The thing was done, and to-night, as for years upon years, that beacon light has shone out upon the ocean and through the storm, and the great fear bas departed, and the navies have sailed secure. Fragile enough looks the structure as it stands against the waves, the foam and the clouds, like an airy phantom, and though there never have been wanting those who have said it shall speedily fall, yet it is fastened to the rock, of material no less imperishable, and whilst generation after generation of noisy men shall come and go, it will still hold on high a sure light which shall guide the sailor in his course as safely as of old time did the pillar of fire the chosen tribes.

Not a hundred years ago and the great question whether men were competent to govern themselves was yet unsolved. The royal Georges and continental despots and diplomats thought not. There were plenty of people who believed that as God had a purpose in everything, and as certain families were good for nothing else, therefore, Q. E. D., they were specially fit for governing. But George Washington, Ben. Franklin, John Adams, John Hancock, Dr. Rush, and sundry others, thought otherwise, and they approved their faith by their works, and for more than three-quarters of a century this nation has shown that some men at least could govern themselves in creditable fashion. When we had fairly begun to believe the problem solved, and that our gubernatorial astronomical system was perfect—along came secession, with its perturbating asteroids, and the swaying of the poles has frightened some of the astronomers of the weaker sort into the belief that our system, after all, is not of the Keplerian and Newtonian kind, but of the effete Ptolemaic shape, and we ourselves not denizens of a mid-day civilization, but twilight Egyptians. Nevertheless, in our firm opinion, it is the vernal and not the autumnal equinox, and the storms accompanying it but herald a more glorious summer than the world of nations has yet seen. The Jeff. Davises, and Lees, and Cobbs, and Beauregards; the Yanceys, and Jacksons, and Floyds are nothing but nebulæ which telescopic cannon will speedily resolve into their original harmless elements.

This light-house among the nations has been anchored upon a sure rock, and the waves of rebellion, anarchy and usurpation, rage and dash harmlessly at its base.

Stability—the fixed point which our minds continually seek, notwithstanding their roaming, speculative tendency—can never exist save upon the ground-work of Truth. The attractions of material things always tend to a common centre, and the weightiest always moves thereto, whatever its occasional resiliencies and reboundings. So in the realms of science, which is but the appreciation by the human mind of its surroundings; all things seek the common centre—Truth. Now and then, from the time of Æsculapius till this day, some men have mistaken the reaction for the attraction; the centrifugal for the centripetal; but steadily and constantly the general mind has been approximating central truth.

When we take this science of Medicine, about which we are just now more especially interested, we find it has precisely the same history with all other things which men think they know. Men's thoughts have been pendulums vibrating to each extreme from central Truth, or they have under the influence of a perpetual tangential force revolved around and around it. Nothing has been more common than for a medical man to be, by the inherent force of his own intellect, prevented from escaping from the influence of central truth, whilst some vile dogma accidentally received has forever kept him in a great orbit at a distance from it. This never has done—this never will do. If you wish to get at the truth of any matter, you must let the mind be free. Its perceptions must be educated and its faculties developed. Its limbs must be unfettered, and no Delilah of dogmatism, or prejudice, or fancy, must be allowed to use her fatal shears upon the locks of its strength. He who lacks confidence in his art can never be a good artist. No impostor even can thoroughly succeed until he first makes a dupe of himself. The man who believes to the uttermost is most truly powerful in the support of his belief. A doubtful cause, or a positive untruth, thoroughly believed in by its supporters will gain headway and a name among men; whilst a truth not at all, or only half believed, will limp and stumble in the race. A whole truth, wholly believed, is the "open sesame" to its possessor, and Ali Baba with all his forty thieves are powerless before its utterance.

Now then—not to be too discursive—young Gentlemen, if you wish to succeed in this art of Medicine which you have come up here to inquire after, you must believe in it, and trust in it, and anchor yourself upon it. When we, your temporary teachers, hold up a beacon to you, you must take care that it is an Eddystone light, and not a will-o-the-wisp, or a wrecker's lantern. When we present to you the principles of medicine, we want you to test them as earnestly, as sagaciously, and as cautiously as you would paper money—I would say coin, but the idea in these times would be too far-fetched and ludicrous.

We wish you to believe implicitly what we shall say to you in the ensuing series of lectures; not because *we* say it, but because it is the solid truth, upon which you can stand and bid defiance to the scarlet women of quackery, and all the regiments of medical delusion with their parti-colored uniforms and caps and bells.

Some things, and the fewer they are the more shall we rejoice, you will have at present to take upon trust with a like generous confidence you extend to Eastern bank notes, or occasionally even Illinois money, but we shall strive generally to give you Treasury notes, drawing not less than 7 3-10 per cent., guaranteed by the Republic of Science. They represent pure gold, but you must recollect that gold is scarce in war time, and the disciple of Æsculapius is a sworn soldier for all terrestrial time against ignorance and deceit, pretense and fraud.

*Oportet discentem credere—oportet edoctum judicare*—it is your duty to place confidence in what we tell you, where you have not full possession of the facts which warrant you in coming to a different conclusion from what we do; where you have full possession of these facts, you may judge. Medicine and Surgery are largely made up of the results of experience, which you have not, but where, as in many parts, they are founded on positively developed principles, you have no choice in the matter, you must accept them.

Putting the whole truth in a nut-shell, we may remark that to-day the larger portion of what you have to learn, during the next sixteen weeks, consists of facts as incontrovertible as the Ten Commandments, or—the Constitution of the United States.

You may have heard about disputes among medical men. It is the favorite theme of empirics and satirists. Learn now, if you have not before, that these disputes and differences of opinion do not touch the pith and marrow of the matter. It is a fact of history that the great wars of the world have occurred in consequence of questions of boundary and not with reference to the great central territory. Little things, outside of the great moving springs of commonwealths, have given rise to commotion and all confusion. And it is precisely so in Medicine. The great central territory of Medicine cannot be invaded. Nine-tenths of what we shall have to say to you is of that character that no debate can arise. If you, or any of you, have come here with the conviction that you must, as a matter of form, attend medical lectures to get a diploma, so as to be candidates for reception of official insolence as army surgeons, or poor pay and hard work as private practitioners, and that it is really not a matter of much importance whether you learn anything or not, as the next decade will overthrow the majority of it at the best—I beg of you to disabuse yourselves of the error just as speedily as possible, for I assure you it is a stupendous hallucination.

Here is my friend, the Prof. of Anatomy, who will convince you that there is positively no question about the sphenoid bone or the circle of Willis; that the cardiac orifices are not openings for debate; that the extensors and flexors have quite other relations than to the manual of arms. He will convince you that the arteries are quite positive in their distribution, and that the veins are contrived to take up something else than streams of argument.

The Professor of Chemistry will demonstrate to you that, in his department, you have something else to deal with than gas of the metaphorical sort. That material substances are put together very ingeniously and unmistakably; that their taking apart involves something more than the wit of an epigrammatist, and that other things besides men and donkeys are "fearfully and wonderfully made." He will show you that the ponderables, whether solid or fluid, have to be mentally digested, and that the imponderables must be duly weighed. It won't do to tell him, by and by, that there is a difference of opinion upon this point, and that really you have not made up your mind.

And then my friend on the right—he who victimizes frogs and dogs, guinea pigs, rabbits, cats, *et id omne genus*, it will never do to say that his branch is very amusing and entertaining, and that on the whole you would pay attention to it, only that you design being a surgeon instead of a *savan*, and a doctor rather than a physiologist. He will prove to you that you can neither be the one nor the other, worth the name, unless you have fathomed not only the mysteries of anatomy—the organs in repose—but also of physiology—those of the organs in action. He will tell you in the lecture room, as he has frequently told me out of it, that every hour of the day in his practice, which all of our citizens know to be immense in extent, he finds that the facts of physiology are sure guides to his judgment; that they give confidence and power, and that without them he would feel like, what he hates more intensely than most men have capacity—an empiric and quack.

And then Materia Medica—he of the bottles and gallipots, the dried specimens and moist abominations, you will be most wretchedly deceived if you fancy that he will let you off without knowing about the armamentaria—where the tools come from, what they are, how to tell the good from the bad, how they individually affect the body in its different conditions of health and disease, when to use and when to avoid. All these things are quite other than speculative, as I believe my learned brother will fully expound to you.

A large part of the practitioner's duty will come under the purview of our friend on the left, who will convince you that the second part of the primeval curse and blessing involves his branch as a necessity, and that ninety-nine hundredths of his teachings are, not only unquestioned but, unquestionable truth. If you fail to apprehend them, and hoard them up in your memory—the time probably will come, even in this life, when, if you have a grain of human pride and sensibility, you will fervently pray for the rocks to fall upon you, and the hills to cover you out of sight.

In the series of lectures upon Surgery, Pathological and Operative, just now, there is especial interest. I do not need to urge it upon your attention—the times urge it upon you. The tide of war leaves wrecks of the human form all along the shores of civil life. But I beg your earnest study here, of those conservative methods which are distinguishing the surgeon of to-day, from him of the books and of past time. I violate none of proprieties of the occasion, when I assure you that in these halls you will be taught principles and methods, in this magnificent branch, brought fully up to the level of the hour.

There has gone abroad among the populace, an impression that a surgeon is emphatically a kind of animal, who lives and moves, and has his being by the knife. Limbs have been improperly sacrificed, and hence there is a tendency to the other extreme so that, instead of limbs, lives are now too often placed in jeopardy. It is trusted that you will hear and comprehend, and subsequently put in practice, precepts which shall render you, as surgeons, conservative of *both* lives and limbs. And you will learn that wielding the knife is but a limited portion of the surgeon's duty—and yet sometimes it is an inexorable duty. Some of you perchance think that all you need to know in order to be good surgeons, is, anatomy and certain mechanical methods. I beg your pardon, but it's a deplorable error. You will find that you cannot even be a good surgeon without thorough mastery of the curriculum of medical study. A man may be a good practitioner of medicine, without being skilled in operative surgery; but no man can be a whole surgeon without being a thoroughly educated physician. I do not hesitate to say that the grandest triumphs which crown the surgery of the present time, have been achieved by recognition of this cardinal truth. There is an *eclat* about the exploits of the bold handler of the catling, saw and scalpel, very attractive to the student, but he must ever recollect that great success is the constant attendant only of great attainments. A guerilla can make a dashing raid, but great victories belong to great generals who make battles not accidents, but assured conquests by scientific forecast. Here and there, it is to be admitted, advances have been made by happy chances, as a traveler in the desert may be saved from starvation by finding a lost bag of biscuit; but as the best way to ward off hunger is by ploughing and planting, sowing and reaping, so the only sure course to success as a surgeon, is by laying broad and deep the strong foundation of general and extensive knowledge.

It is a tradition among medical students, which antedates the memory of the oldest inhabitant, that the occupant of each chair in the medical college is officially bound to magnify his office. And to-morrow you will hear divers observations from my worthy colleagues, which, I doubt not, will give strong color to the suspicion that this tradition is founded upon fact. Each of them will assure you that his particular department is all-important; that "Ichabod" might be written upon the

Æsculapian temple if his particular pillar were torn away. And, believe me, Gentlemen, the statement of each will be, and is, true and undeniable to the uttermost letter. If some modern Samson in his blind rage should apply his brawny shoulders to any one of these great columns, the whole edifice would tumble into indiscriminate ruin. The curriculum of study requires the support of each branch. The Royal Arch of Medical Science demands the support of each one of these as, reciprocally, keystones to the rest.

The time has gone by when a man can be a good practitioner of medicine or surgery, without general knowledge of these different departments.

The world has kept moving since we were boys, and he who stands still falls in the rear, by the inexorable logic of events, as the politicians love to phrase the idea.

It is but a little while ago that physiology, as a science, was born—it is to be regretted that so many have not even yet recognized the advent of this prophet of an entirely new medical dispensation. For this beautiful science, deny it who may, has changed the practice of medicine from foundation to capstone.

Observe now—you cannot understand physiology unless you are generally and minutely acquainted with anatomy—it is sheer nonsense to think otherwise. Wherever a nerve thrills or an artery throbs, you have got to carry the scalpel and the lamp. You cannot run a steam engine even, save in the most hazardous and break-neck fashion, unless you understand its parts. Do you fancy you can play upon "the harp of a thousand strings" as easily as upon a tin trumpet? Some people appear to think so.

And then, when you have mastered anatomy, you can know nothing of physiology until you have the principles and facts of chemistry as familiar as household words. Mark this, for it is meant—earnestly meant. Perhaps some of you will say —Good Heavens! the human system is not a laboratory, nor the stomach a Papin's digester. Some of you may have read, in some antediluvian book, that chemistry has nothing to do with the animal body. I pray you get rid of this idle fallacy —it is one of the devices of the great adversary of bodies. You will hear something about the correlation of physical and vital forces. You will hear that the human organism, however much or however little soul it has in it, is made up of material atoms, and all forms and shapes of matter and force operate within and around it, and you can have no idea of what either matter or force may be, unless you cultivate the closest intimacy with the chemist and all his formidable array of retorts, spirit lamps, test tubes, gasometers, electrical apparatus and batteries. Let me assure you, chemistry is everywhere a necessity to the medical man ; you might as well undertake to study and practice medicine without eyes or ears, as without anatomy or chemistry.

And here permit me one little word—Prof. Blaney, so long identified with our college, the present session will be absent from us. He is using his varied and extensive professional acquirements in the service of the national government. Some of you will miss his familiar face and genial smile. For " none know him but to love him, none name him but to praise." Ranking as a man of science second to no other American in his particular department, those of us who know him almost lose sight of his learning when we recall his personal qualities. We half forget the scholar and physician, in the accomplished gentleman and always frank and cordial friend. May all the gods of war and peace preserve him from the perils of the field and camp and hospital ; and may Abraham Lincoln & Co. see to it that his patriotic sacrifices may be crowned with something more tangible than glory.

The personal presence of the distinguished gentleman who is to fill the chair of chemistry, in Dr. Blaney's absence, prevents a word about him. The speaker may merely observe, that having himself attended two full courses of his lectures on this branch, he was particularly anxious, and is thoroughly content, to have our Elijah's mantle fall exactly where it has.

Thus far, nothing about Pathology, and hence a word must be thrown in. The whole world of medicine, not to speak of "the rest of mankind," assumed for ages that disease was something erratic, eccentric, or anything but an orderly phenomenon. Mythologists made it depend upon the arrow of some capricious god, and speculatists of all sorts, Christian, pagan and infidel, have fancied a multitudinous host of ghastly phantoms clasping each his particular dart ready to launch at poor victims; or again have depicted earth, air and sea full of grim messengers of death, each ready, vampire like, to seize upon specimens of mortality—candidates for immortality. That which was but a figment of poetry or mythology, unfortunately was translated into the terrible prose of common practice. The attempt was to deal with diseases as though entities—enemies to be met with specific weapons—devils to be fought with fire. They were to be tortured out of the body in which they had gained lodgement. Some people thought they would be soonest exorcised, by agents which would produce precisely opposite effects upon the worried organs. Others thought that they were solitary, inharmonious demons, and the way to drive off one was by effecting the lodgement of another. By this shrewd strategic movement, it was believed the original hostile demon would find it a corporeal and spiritual necessity to "change his base." Another set believed, and even now cherish and trumpet the belief, that, like people of the same trade and opinion, the nearer they are alike, the less they can agree; hence if you can get up a disease as near as possible like the original one, and yet not identical with it, the greater the chance of the dislodgement of the devil in possession. This, it is to be confessed, sounds like satire, but these are among the facts of history.

Some men positively believe that heat is life and cold is death; others think that—

> "Disease is dirt; all pain the patient feels
> Is but the soiling of the vital wheels"—

hence water is the sovereign panacea, and Father Matthew the modern Hippocrates.

Others think that if the patient is sick, it is evidence enough that he is not suitably "Biologized"—if you know what that means, in Heaven's name tell us!

We have not time even to glance at the vagaries which have been indulged, by men in the profession, and men and women out of the profession—we only allude to them to usher in the plain proposition that practical medicine is not a science of mysteries—it has nothing of the mysterious about it. It is a plain common sense affair, in which, perhaps, one man may excel another, precisely as one painter may excel another, not because of any occult virtue in the colors he employs, but because, as Mr. Opie says, he mixes them with *brains*. You will get your colors here; you will mix them with brains yourselves. If you can't do that—God help you!

When old Naaman the Syrian took a long journey to consult the celebrated Hebrew practitioner, he was terribly disappointed because the prophet did not mesmerize the leprous spot—making passes over it and invoking the *Dii Superiores* with potencies of infinite spiritualization,—and plenty of people in these days refuse all credit to medical practice which does not come up to Naaman's notion. The human mind dearly loves a mystery, and is half inclined to think, with Mr. Toots, that what is not mysterious is "of no consequence—none whatever." Dipping in Goose creek or the Chicago River certainly can neither of them be considered cleanly and desirable, but even this may rid some men of worse troubles. But tell a patient to swallow a compound as nauseous, if possible, as either, and he will do it with joy unspeakable.—Fifteen minutes before putting down this identical sentence, I read in the Chicago *Tribune* that Dr. Scovendyke, of the U. S. Hospital service, has discovered an infallible remedy for camp diarrhœa—what think you it is? Substitution of good food properly cooked, and good regimen for utter recklessness of habit? O no! *goose's gizzard!*—or any other fowl. Incidentally, Dr. Scovendyke remarks, that plentiful exhibition of opium and sugar of lead should accompany the gizzard.

O Dr. Scovendyke—Dr. Scovendyke, thou art the representative man—thou hast pluck to uphold the gizzard!

The statistics of English and continental military service show that the health of the soldiery is easily maintained upon a par with that of civil life. The question will recur—is it because the surgeons make constant use of goose's gizzard, or some other natural or supernatural agent? Or, by the baldest hypothesis, may it not depend upon the better observance of the laws of health and life? Will goose's gizzard have the effect in old time attributed to horse shoes nailed over the door? Or is it possible that hygiene has something to do with the matter?

This thing of goose's gizzard has wider relations than appear on the face of it. Dr. Scovendyke has opened a vein.

Order is not only Heaven's first law, but Earth's first law. Pathology is not an exception. The organs of the body, from the simplest cell up through muscle and gland to brain and spinal cord, act ever in an orderly manner. Change the conditions in which they are placed, and you change their action; but ever they act in accordance with the law of ORDER. Pathology is but Physiology with changed conditions of action.

Simple as the decalogue, as true as biblical promises. The study of Pathology, then, engrafts upon Physiology simply the study of the new conditions in which the organs are placed.

All of the practice of medicine, as in fact all of the practice of surgery, consists simply in restoring the conditions which Physiology in its widest import points out as necessary to the normal order.

The grand difference between the practice of medicine today and that of old time is, that now we seek to restore the conditions of health; then they sought specifics, panaceas, and Morrison's Pills;—except now perhaps some of us substitute Chlorate of Potash, Veratrum Viride or goose's gizzard. They used to beat the gongs and blow the hugags to drive away eclipses, with a *post hoc ergo propter hoc* argument exceedingly favorable to repetition the next occultation. Astronomically that music is "played out," but now and then you will find floating through practice in the medical world a similar waif from antiquity. This is illustrated in what is called "perturbating" treatment. You do not know what to do for a patient, or you have failed heretofore; according to this delectable system you must go to work to make him so much worse, that if he chances to recover from it, he shall believe himself about well from strong contrast. I need not say that in the light of modern physiology and pathology, this is simply—egregious folly.

Men are very much alike, and the influences which operate upon them are very similar, hence the derangements to which they are subjected have strong resemblances. These resemblances occurring often in groups, it is convenient to designate these groups by particular names, but it must be recollected that these names are wholly arbitrary—they have no foundation in nature. Take even the most striking groups, those produced apparently by a specific poison, small pox, measles, scarlet fever, etc., etc., and the whole medical world knows, or should know, that the so-called disease is not a uniform phenomenon. Take Typhoid Fever, Pneumonia, Hepatitis, Delirium Tremens, Phthisis, Rheumatism; years ago it was supposed that diagnosing, or naming, these affections was sufficient to indicate the treatment. To-day the veriest tyro knows (or if he does not know, ought to be basted for his ignorance) that the name is by no means a sure index of treatment. The Magnetic needle ought to point due North, but it does not point North ever, and sometimes indeed it points in almost any other direction. But this is not a fair illustration, for the needle is vastly truer to the pole than is the name of a disease to its treatment.

I know this opens a capital loop-hole to idleness. Some men will say there are lions in the way, and will wait till they leave—about as endless a waiting as for the flood of the river to run by. But this will never do. It is easy to say you will treat diseases upon "general principles;" but what are your "general principles?"

The time has gone by when, compendiously enough, you can bleed and give calomel for what is called "inflammation," a term which, like charity, has covered a multitude of sins. The time has not even come when Veratrum Viride or Tartarized Antimony may be substituted. You have got to mix these things, and many others, with brains, in order to give proper color to your claim to practice medicine. Goose's gizzard won't answer.

You will find that the world has moved away even from much that is in your text books. The most that is in them still stands and will stand forever, but speculations, and dogmas, and fancies, and hypotheses, which yet do too much abound in medical works, grow small by degrees and beautifully less as the years move on. Cullen's "spasm of the extreme vessels" and Cullen's treatment of diseases have in great part passed into the Beulah, the beatitude of practical historical oblivion; but Cullen's descriptions of what he actually saw, are as vivid and truthful now as ever. The "archeus" of Van Helmont has been dethroned, and the "morbid spissitude" of Boerhaave has been dissolved, but the greater part of what these keen observers set down is recorded for all ages.

Opinions, and doctrines and systems fade away, but facts and the laws legitimately induced from them can never be overthrown. You may wrongly interpret the fact and to you it is therefore essentially a falsity, but nevertheless you must recollect that the truth, as such, is wholly independent of your or my reception of it. Truth scarcely needs logic to sustain it; indeed it may be asserted, almost without reservation, that whatever requires fine-spun and long-drawn argument to convince, is a little more likely to be false than true. Good eyes are achromatic, but it is easy to apply to them various mechanisms which shall distort everything around, and cover the most transparent truth with a prismatic halo of falsehood.

A good sound fund of common sense, an article popularly well understood, but which all the metaphysicians have failed satisfactorily to define, is worth in our profession whole acres of that morbid mental constitution so often supposed to be genius. It is a melancholy fact that so-called geniuses in medicine are, ten to one, the worst practitioners extant, and their teachings are about as useful and reliable as the sheet iron thunder of a theatre in a dry season. This very useful commodity, common sense, is worth assiduous cultivation. So far as the practice of medicine is concerned, I can assure you that an investment in that stock will be worth more to you than—the perusal even of Paine's big volumes on the fossiliferous strata of medical bibliography and learning.

But remember that common sense in a head without knowledge, is like the precious jewel in the head of the ugly and venomous toad—it is power without an engine—gold in the pockets of a Selkirk—spurs and a standing collar to the king of all the Mosquitos. Keep what common sense you happen to have by you; get more if you can, and then acquire all stores of knowledge. This your common sense will fashion into enginery and instruments which shall compel the elements to be your servitors.

Do not lose sight and memory of the slightest fact bearing upon the science. At the best you will learn too little. The fact which perhaps now seems trivial to you, by and by, in the chamber of sickness, or of study, may supply the last link in the chain of your discovery—it may prove the sure clue to the labyrinth of disease and its remedy. The little motion of the needle gave a new world to Columbus and mankind. The imprisoned forces of a water drop may explode the mined magazine, bring back life from the grasp of death, may determine a nation's existence or a despot's doom. One little wire laid upon another and then another, and so on, and on, has spanned the abyss of Niagara so that great locomotives and gigantic railway trains sweep over it in perfect safety, though hung at a dizzy altitude in air. A little touch upon a black grain, not so big as the head of a fairy's bodkin, and a hundred pound ball goes hurtling through the atmosphere, swifter and more destructive than the thunderbolts of fabled Jove.

There is said to be a point in the animal nervous centre where if you insert a needle, a little way only, life vanishes. And thus all knowledge is made up of littles, and he who scorns them can never be truly wise.

The fact is, attention to the little things constitutes the grand distinction between the good and the poor practitioner. A vast number of men can perform surgical operations even of the most capital character, but their success will be the most diverse in degree. One, content with a brilliant use of the knife, which he handles as lightly and daintily as a writing master flourishes the quill, will operate to the admiration of all bystanders, but, while the operator recovers, the patient dies, simply because he does not attend to those little circumstances which determine, first, the propriety of the operation, and, second, by judicious after-treatment secure its success.

The popular mind is dazzled by the off-hand bluff decision of a "heroic" practitioner, who now and then removes disease by a magnificent *coup d'etat*, but the profession have learned, by grim and sad experience, to distrust the architect of great cures. The triumphant Garibaldi of one campaign, speedily is transformed into the defeated, wounded, imprisoned and broken-hearted Garibaldi of the next.

Be assured you can not work miracles, although the apostles of some false god undertake to persuade you that the day for miracles has not yet passed.

You will succeed, if you succeed at all, by patient study and constant accumulation of truths. Not that popular success will wait upon your knowledge—that by no means follows—for popular success is just now more likely to be the meed of the charlatan, the quack and the knave, than that of the true scholar. But the eventual reward is certain. Burglary is sometimes easier than honest toil and the profits more immediate and abundant, but I am not here to advise you to take that up as a branch of business. The first element of success is that you really deserve it. And the way to deserve it is, not by cramming from a manual, or stuffing for an examination, whereby you may possibly outwit even the Examining Board, but by steady, straightforward gathering up and digesting of solid knowledge.

We are fallen upon strange times. Two years ago and had any one predicted the things which we now see about us, he would have met the fortune of Cassandra at old Troy. None would have believed, and none would have paid the slightest heed.

Two years ago and the medical profession was tolerated. Legislatures and all the powers that were, looked upon us as merely an incidental appendage, and not as a necessity of society. Political charlatans thought to gain ascendancy by fostering the bastard scions of the medical tree. Medical men, relying upon the impregnability of their position, like Major Anderson at Sumter, allowed the hordes of quackery to establish batteries all around them. The fiat had gone out from headquarters—bear and forbear. The laws did not protect us and demagogues attacked us. Pitiful whipsters satirized us; spiritual tables thumped against us; homœopathists annoyed us as the fly did Uncle Toby. The country was full of people; population was a drug, and Malthus was invoked against Hymen. Horses and cattle were considered valuable property, and agriculturists took especial care of them. Men were at a discount; children a misfortune, and women only ornamental.

To-day things are quite different. The country recognizes the value of men. The Lilliputians have sent up squeak after squeak of rage; but the country recognizes and acknowledges its want of medical men,—not shams, not etherealized nonentities, or wooden-headed knaves. Population has ceased to be a drug, and men with sound minds and healthy bodies are admitted to be the sinews of war. Examining Boards, too often the offspring of political adultery, and the creatures of emasculated cliques, have done what they could to prevent fulfillment of the national want, but to-day the medical man stands the peer of the proudest general, and to his skill more than to the boasted strategy of the military schools is success or defeat attributable.

The bayonets and bullets of battle slay infinitely fewer than the vile hygienic arrangements, and unnecessary villainy of the camps. To conduct this war to a successful termination, discreet and sensible physicians are worth more than all the great generals, the military celebrities and geniuses the crisis has yet spawned.

I do not speak loosely or unadvisedly—the statistics are before the world. The great element of success is men, healthy, strong men, and in the dark and gloomy night of war which is upon us, the medical profession, more than any and all others, are the arbiters of the nation's destiny. I throw down the glove to any who dares dispute this primal fact. The surgeon of a regiment has an infinitely more responsible duty than its colonel, and that he should be ranked by the latter, is a paradox which future ages will look upon with unmitigated astonishment and disgust. More medical men, proportionately, than of any other class have fallen victims of the war, and to-day more are willing to assume the perils of the camp and field.

Many of you, I know, are preparing for a part in this great struggle for national existence. We, of the Faculty, will promise you our best efforts to suitably prepare you for this vast responsibility. We ask you to be sincere and earnest and industrious. We shall not decive you by the idea that we have possession of miraculous amulets against disaster; that we have panaceas, whether Calomel, Veratrum Viride, Chlorate of Potash, or even Quinine and Goose's gizzard to present you with. We shall endeavor to explain to you the physical, and, to some extent, the mental structure of man, and the varying influences which operate upon him. With the first Napoleon we say, " the tools to him who knows how to handle them." We shall do our best. We only ask that you shall do yours.

We trust that in one thing you may be disappointed—that before the brief session now before us is ended, this deplorable strife may be over; that the curtain may fall upon this fearful drama; not upon the 'shattered fragments of a once glorious but now dissevered Union,' but upon the more magnificent tableau of the great family of States, hand in hand, destined to be "the praise and heroic song of all posterity."

We look for peace—honorable and glorious. But whether it comes sooner or later, you will find that the medical profession will emerge from the conflict crowned with laurels heretofore denied, but honorably won.

There is not one of your present instructors who would not, long before this been in the ranks of the grand army of the Union, if he had not believed that duty required him to take the part of preparing those for the conflict whom the nation needs more than it needs arms or munitions of war.

We have thus a double duty: to prepare you for the gentle and kindly duties of peace, or for the stern and fearful demands of the war.

On our part, we pledge again our best efforts. On yours, we ask but a realization of your duty and responsibility. To whatever of assistance we may afford you in the work now at the door, on behalf of my colleagues and for myself—I bid you cordial Welcome!

